# Multiscale Interactome-Guided Discovery Candidate Herbs and Active Ingredients Against Hyperthyroidism by Biased Random Walk Algorithm

**DOI:** 10.3390/ijms26199789

**Published:** 2025-10-08

**Authors:** Seok-Hoon Han, Ji-Hwan Kim, Yewon Han, Sangjin Kim, Hyowon Jin, Won-Yung Lee

**Affiliations:** 1 College of Korean Medicine, Wonkwang University, Iksan 54538, Republic of Korea; 2 Department of Sasang Constitutional Medicine, Division of Clinical Medicine, School of Korean Medicine, Pusan National University, Busan 46241, Republic of Korea; 3 National Institute for Korean Medicine Development, Seoul 4516, Republic of Korea; 4 Department of Korean Internal Medicine, College of Korean Medicine, Wonkwang University, Iksan 54538, Republic of Korea; 5 Research Center of Traditional Korean Medicine, Wonkwang University, Iksan 54538, Republic of Korea

**Keywords:** hyperthyroidism, herbs, active ingredients, multiscale network

## Abstract

Hyperthyroidism features excess thyroid hormone and a hypermetabolic state; although drugs and definitive therapies exist, mechanism-anchored options are still needed. We built a multiscale interactome and applied a biased random-walk diffusion model to prioritize herbal candidates, active ingredients, and mechanisms. Herb–compound records came from OASIS; targets from DrugBank, TTD, and STITCH; and disease genes from DisGeNET. For each herb and compound, we simulated diffusion profiles, computed the correlation with the hyperthyroidism profile, and assessed target overlap ratio. Herbs were ranked by correlation and *p* < 0.05 overlap, retaining those with ≥5 active compounds linked to disease targets. Top signals included *Geranii Herba* (0.021), Gastrodiae Rhizoma (0.012), and *Veratri Rhizoma Et Radix* (0.011), plus seven herbs at 0.010. Herb–disease relationships were strongly enriched. Enrichment analyses highlighted MAPK, PI3K–AKT, p53, HIF-1, and thyroid hormone signaling, with Gene Ontology terms for apoptosis/anoikis, inflammation, and RNA polymerase II-dependent transcription. Compound-level analysis recovered evidence-supported ellagic acid and diosgenin and proposed resveratrol, cardamomin, 20-hydroxyecdysone, and (Z)-anethole as novel candidates. Subnetwork mapping linked these compounds to phosphorylation, GPCR–cAMP/TSH signaling, and transcriptional control. This framework recapitulates known thyroid-modulating herbs and elevates underappreciated leads with testable mechanisms, supporting the discovery of multi-target therapeutics for hyperthyroidism.

## 1. Introduction

Hyperthyroidism is a condition characterized by excessive production and secretion of thyroid hormones, leading to a hypermetabolic state with symptoms such as fatigue, weight loss, heat intolerance with increased sweating, palpitations, anxiety, and insomnia [1]. Even in iodine-sufficient countries, the prevalence of overt hyperthyroidism has been estimated at 0.2–1.3% [2]. Graves’ disease—an autoimmune disorder driven by thyroid-stimulating antibodies targeting the thyrotropin receptor—is the leading cause of hyperthyroidism [1]. Serum thyroid-stimulating hormone (TSH) is the most sensitive and specific initial test for diagnosis, complemented by measurement of thyroid hormone levels (T3, T4) and assessment of disease-specific autoantibodies when appropriate [3]. Standard treatments include antithyroid drugs, radioactive iodine ablation, and thyroidectomy, selected according to etiology and patient factors. However, long-term antithyroid drug therapy carries risks of adverse effects, and definitive therapies can result in hypothyroidism and other complications [4,5]. Safer and more effective therapeutic options are therefore still needed.

Herbal medicine—with its multi-component, multi-target modality—has attracted attention as a complementary strategy for complex thyroid disorders. Historically, seaweed-derived materia medica such as *Haizao* (Sargassum) and *Kunbu* (Laminaria) were prescribed for conditions analogous to goiter, aiming to soften indurations and resolve phlegm stasis [6]. Clinically, Haizao Yuhu Decoction (HYD) has demonstrated symptomatic improvement and reduced relapse compared with methimazole in patients with Graves’ disease, suggesting utility for individuals intolerant to antithyroid drugs [7]. Reflecting these observations, expert consensus in China recommends iodine-containing herbal prescriptions for patients with hyperthyroidism who are intolerant of antithyroid drugs or decline radioactive iodine or surgery [8]. Mechanistic and preclinical studies further indicate that Sargassum fusiforme polysaccharides have demonstrated antioxidant and immunomodulatory activities in vitro and preclinical models; notably, enzyme-hydrolyzed forms enhanced free-radical scavenging and immune activation in RAW264.7 cells [6,9]. Collectively, these findings support the multi-target therapeutic potential of herbal medicine in hyperthyroidism and motivate systematic discovery of safer candidates with clarified mechanisms.

Despite encouraging clinical and experimental data, the molecular mechanisms underlying herbal efficacy remain incompletely defined, which hampers evidence-based adoption and translation to drug discovery [10,11]. Advances in systems biology have catalyzed network pharmacology approaches to dissect the complex interactions among herbal compounds, protein targets, and disease pathways [12,13]. Such methods enable system-level visualization of drug–target–disease relationships and prioritization of key mediators. Prior studies have leveraged network pharmacology to elucidate active compounds and pathways of *Morus alba* against diabetes and to map the multi-pathway actions of Xiaochaihu Decoction in non-alcoholic fatty liver disease [14,15]. More recently, multiscale interactome frameworks combined with biased random walk algorithms have been proposed to model how drugs perturb disease contexts and to identify promising herbal candidates and active ingredients [16].

**Figure 1:**
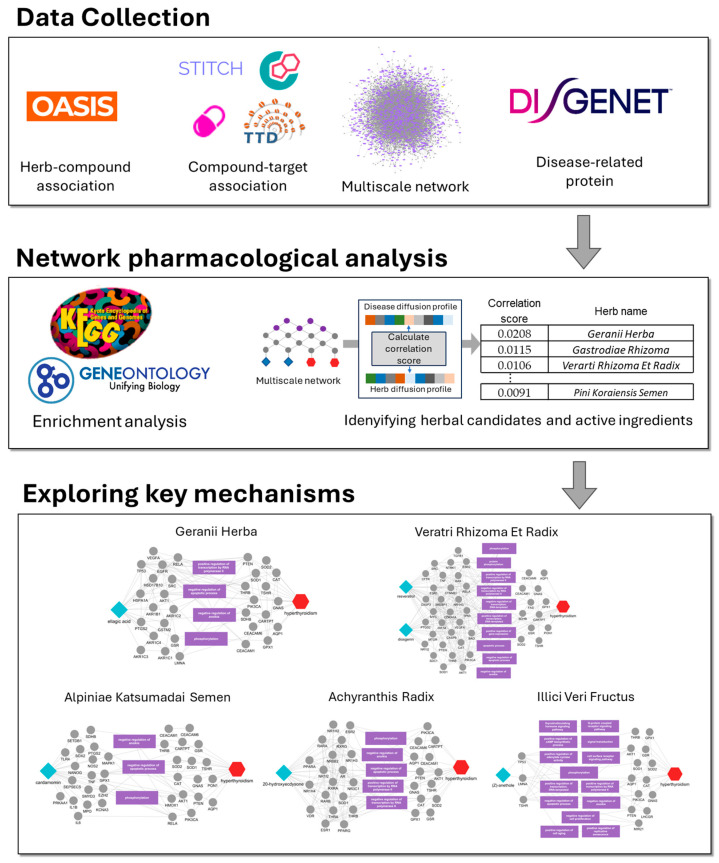
Schematics for identifying candidate herbs and active ingredients for hyperthyroidism. This schematic illustrates the use of multiscale network analysis to identify herbs and active ingredients with potential efficacy against hyperthyroidism. Herb–compound and compound–target associations were mapped, and disease-related proteins were identified. Diffusion profiles for herbs and disease proteins were calculated and compared, prioritizing herbs with high correlation scores. Enrichment analysis revealed key biological pathways, while individual ingredients of top herbs were further analyzed to highlight core protein targets. The bottom panel shows multiscale-level mechanisms for selected herbs.

In this study, we aimed to systematically identify herbal candidates and their bioactive compounds with potential therapeutic relevance to hyperthyroidism and to elucidate their molecular mechanisms using a multiscale interactome-based network pharmacology framework. We first constructed an herb–compound–target knowledge base and curated hyperthyroidism-related disease targets (Figure 1). We then embedded these data into a multiscale network and executed a biased random walker algorithm to compute disease and herb diffusion profiles. By correlating herb-specific profiles with the hyperthyroidism disease profile, we prioritized herbs with the strongest disease relevance. We further identified key protein targets and enriched biological pathways regulated by these candidates. Finally, we extended the same random walker-based analysis to constituent compounds of top-ranked herbs to nominate disease-relevant active ingredients and infer their mechanistic roles. Taken together, this multiscale framework offers a principled route to nominate multi-target herbal therapeutics for hyperthyroidism and to link candidates to pathophysiological mechanisms.

## 2. Results

### 2.1. Identification of Candidate Herbs for Hyperthyroidism

To identify herbs with potential efficacy against hyperthyroidism, we first curated herb–compound records from the OASIS herb database. We then mapped these compounds to protein targets using validated resources, including DrugBank, TTD, and STITCH. Using the compiled herb targets, hyperthyroidism-related disease targets, and proteins/biological functions embedded in the multiscale network, we applied a biased random walk algorithm to compute diffusion profiles. Similarity between each herb and hyperthyroidism was quantified as the correlation score between their diffusion profiles. We additionally assessed the significance of target overlap between each herb and the disease using a hypergeometric test.

Herbs were prioritized if they showed both a high correlation score and significant overlap with disease targets (*p* < 0.05). From these, we selected the top 10 herbs that also possessed ≥5 active compounds with significant associations to disease-related proteins. For these top herbs, enrichment values ≥5 were observed for herb–disease target relationships, indicating that the multiscale, diffusion-based model successfully recovered disease-relevant targets The top set comprised *Geranii Herba* (0.021), Gastrodiae Rhizoma (0.012), *Veratri Rhizoma Et Radix* (0.011), and seven herbs tied at 0.010: *Laminariae Japonicae Thallus*, *Alpiniae Katsumadai Semen*, *Achyranthis Radix*, *Illici Veri Fructus*, *Ligustici Tenuissimi Rhizoma et Radix*, *Mori Folium*, and *Pini Koraiensis Semen* (Table 1). Herb–disease target relationships were strongly enriched (enrichment 16.9–52.8), and literature support was present for *Geranii Herba*, *Veratri Rhizoma Et Radix*, and *Laminariae Japonicae Thallus* (PMIDs in Table 1).

Notably, several top-ranked herbs—such as *Geranii Herba*, *Veratri Rhizoma Et Radix* and *Laminariae Thallus*—have prior reports supporting thyroid-modulating activity, consistent with our findings [17,18,19]. For instance, ingestion of Kombu (Laminaria japonica)—an iodine-rich seaweed—transiently suppresses thyroid function (TSH rises with slight FT4/FT3 reduction) and can cause reversible hypothyroidism, consistent with excess iodine exposure [17,18]. In a murine cold-stress model of hyperthyroidism, arecoline—the major alkaloid of *Areca catechu* seed—reversed hyperthyroid changes (lower T3/T4, higher TSH), indicating thyroid-suppressive activity in vivo [19]. By contrast, other herbs have limited or no prior evidence for hyperthyroidism with a high correlation score and significant protein overlap. These results nominate them as promising, previously underappreciated candidates for therapeutic development.

### 2.2. Herb-Ingredient-Target Network Construction of the Top 10 Herbs

**Figure 2:**
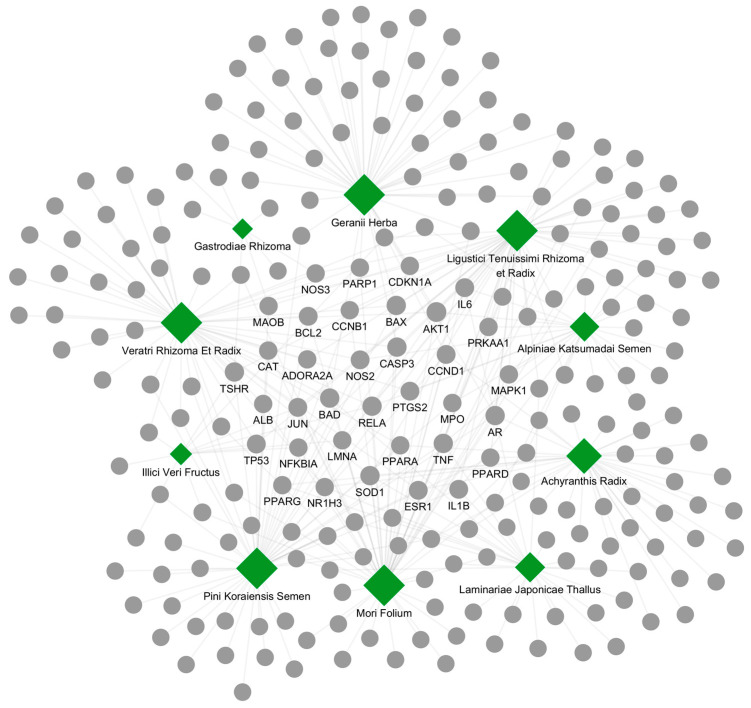
Herb–target interaction network of the top 10 candidate herbs with the highest correlation scores for hyperthyroidism. Each green hexagon represents an individual herb, while gray circles indicate the corresponding protein targets. The edge connections illustrate herb–target associations derived from curated interaction databases. Node size (both hexagons and circles) is proportional to the interaction frequency between each herb and its targets, ranging from 1 to 50 connections. Node labels display the 49 core protein targets that are shared by three or more of the 10 candidate herbs.

We next constructed and visualized an interaction network linking the top 10 herbs to their protein targets (Figure 2). The network comprised 10 herb nodes, 95 target nodes, and 178 herb–target edges. This structure illustrates the multi-component and multi-target nature of these herbs and their potential to modulate complex disease biology. Only 28 proteins were targeted by ≥3 herbs, highlighting a restricted set of putative core targets. In particular, TP53 was shared by nine herbs; AKT1, MAPK1, and IL6 by eight herbs; and TNF, CASP3, and MAPK3 by three herbs. These shared nodes likely represent mechanistic hubs through which diverse herbal constituents converge to exert therapeutic effects in hyperthyroidism.

### 2.3. Enrichment Analysis of the Top 10 Herb Targets

To further interrogate the core protein targets of the top-ranked herbs, we performed enrichment analyses using KEGG pathways and Gene Ontology. KEGG analysis revealed significant associations with pathways implicated in thyroid pathophysiology and stress signaling, including the MAPK signaling pathway, Thyroid hormone signaling pathway, Calcium signaling pathway, and HIF-1 signaling pathway. Additional disease-relevant pathways—PI3K–Akt, MAPK, p53, and HIF-1—highlight processes linked to cell survival and death, immune modulation, and oxidative-stress responses, alongside the thyroid-specific Thyroid hormone signaling cascade (Table 2).

**Figure 3:**
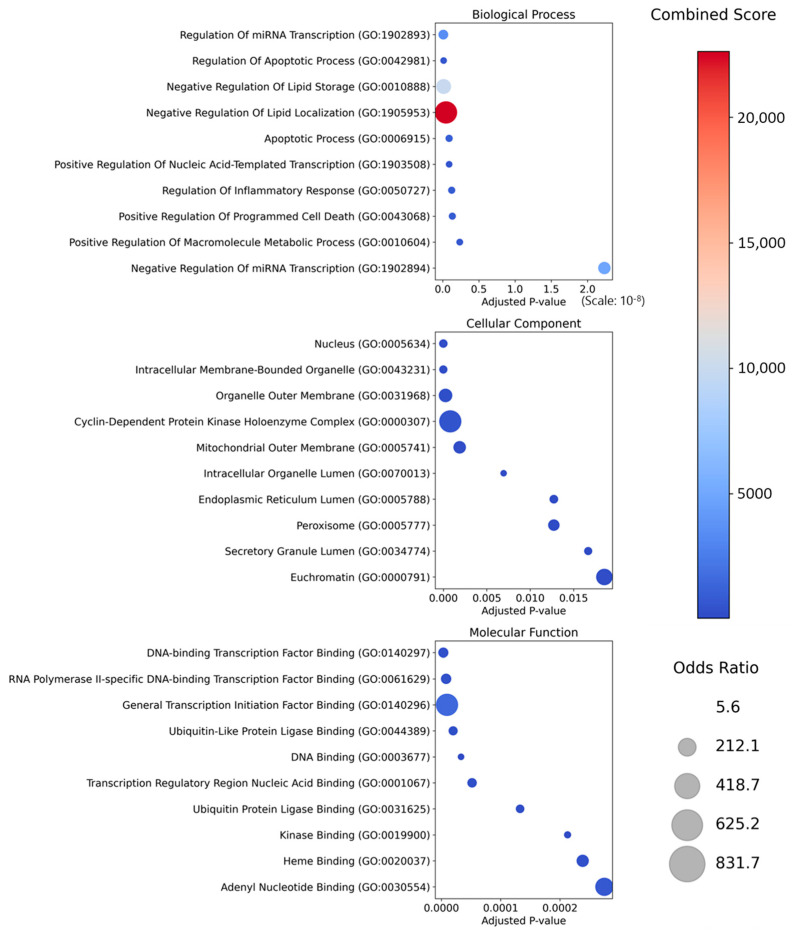
Gene Ontology Enrichment Analysis of Target Proteins from Prioritized Herbal Candidates. This figure shows the GO enrichment analysis of target proteins related to prioritized herbs for psoriasis treatment, categorized into Biological Process, Cellular Component, and Molecular Function. Each bubble represents a GO term, with its size indicating the odds ratio (enrichment strength), and its color reflecting the combined score (based on *p*-value and odds ratio). Combined Score: Darker red indicates higher enrichment of the GO term with the target proteins. Odds Ratio: Larger bubbles suggest stronger associations between the GO term and the targets.

GO enrichment supported these findings (Figure 3). In Biological Process, terms related to apoptotic regulation, inflammatory response, and oxidative-stress control were over-represented. Cellular Component analysis showed that most targets were located in the nucleus, mitochondrion, endoplasmic reticulum, and peroxisome. This localization pattern is consistent with roles in intracellular metabolism, protein synthesis and folding, and redox homeostasis. In Molecular Function, targets were enriched for transcription factor binding and regulation of RNA polymerase II-dependent transcription, suggesting their potential influence on gene-expression programs. Additional functions included ubiquitin ligase binding (protein turnover), kinase binding (signal-transduction control), and heme binding (redox and energy metabolism). Collectively, these enrichment results indicate that the prioritized herbs converge on signaling and functional modules central to hyperthyroidism—providing mechanistic anchors through which multi-component interventions could modulate disease biology.

### 2.4. Identification of Potential Active Ingredients and Multiscale Network Mechanism Analysis of Candidate Herbs

We performed a compound-level analysis of five prioritized herbs: *Geranii Herba*, *Veratri Rhizoma Et Radix*, *Alpiniae Katsumadai Semen*, *Achyranthis Radix*, and *Illici Veri Fructus*. This analysis aimed to infer putative mechanisms on a multiscale network. For each herb, compound–target annotations were mapped onto the network, and a biased random walk was applied to compute compound diffusion profiles. Compounds were then ranked by their correlation with the hyperthyroidism disease profile. Candidates showing both a high correlation score and significant target overlap with disease proteins (*p* < 0.05) were designated as putative active compounds (Table 3). This analysis identified rutin (0.0217), beta-sitosterol (0.0161), and quercetin (0.0092) as the active ingredients in Benincasae Semen, each exhibiting high correlation scores with the hyperthyroidism diffusion profile. In Houttuyniae Herba, norisoboldine (0.0473), hyperoside (0.0261), rutin (0.0217), and quercetin (0.0092) showed significant correlations with hyperthyroidism -related targets. Glehniae Radix was characterized by falcarindiol (0.0557), and Corydalis Tuber by tetrahydrocoptisine (0.0846), each as the sole ingredient with both high correlation scores and statistically significant protein overlap within the disease diffusion profile (Table 3).

This analysis nominated ellagic acid from *Geranii Herba* (0.007) as an active compound with a significant association to hyperthyroidism-related targets. From *Veratri Rhizoma Et Radix*, resveratrol (0.013) and diosgenin (0.011) met the selection criteria. *Alpiniae Katsumadai Semen* yielded cardamomin (0.012), and *Achyranthis Radix* yielded 20-hydroxyecdysone (0.009), each showing significant protein overlap within the disease diffusion profile. No additional compounds from *Illici Veri Fructus* reached the predefined thresholds. Collectively, these results highlight discrete constituents that may drive the predicted anti-hyperthyroid activity of the corresponding herbs.

We confirmed that, among the prioritized actives, ellagic acid from *Geranii Herba* and diosgenin from *Veratri Rhizoma Et Radix* have prior reports supporting therapeutic effects in hyperthyroidism. By contrast, resveratrol (from *Veratri Rhizoma Et Radix*), cardamomin (from *Alpiniae Katsumadai Semen*), 20-hydroxyecdysone (from *Achyranthis Radix*), and (Z)-anethole (from *Illici Veri Fructus*) lack direct evidence in this disease context, suggesting that they may represent novel candidate actives with potential benefit. These observations indicate that our multiscale network framework successfully prioritizes both known and previously under-recognized bioactives for hyperthyroidism.

**Figure 4:**
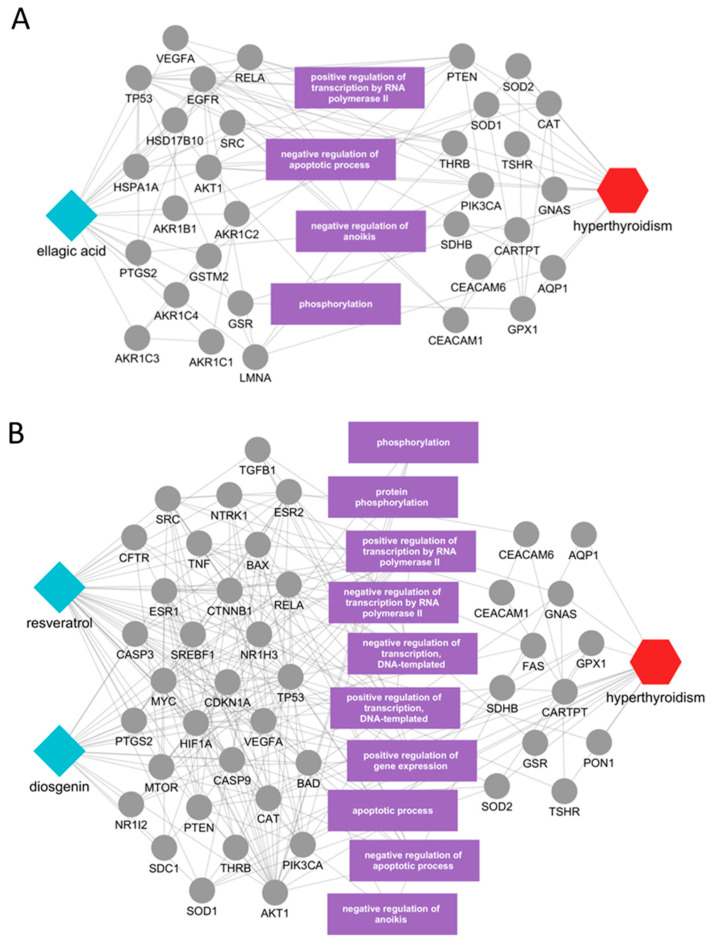
Key mechanisms of herbs with evidence-supported constituents in hyperthyroidism. (**A**) *Geranii Herba*; (**B**) *Veratri Rhizoma Et Radix*. Cyan diamonds represent compounds, gray circles represent protein targets, purple rectangles indicate biological processes, and the red hexagon denotes the disease (hyperthyroidism).

To explore mechanisms underlying these activities, we constructed disease-focused subnetworks that connect each active compound to hyperthyroidism-relevant protein targets and enriched biological functions. For *Geranii Herba*, the ellagic acid subnetwork indicated direct links to disease-related proteins, including THRB, TSHR, GNAS, EGFR, AKT1, PTEN, SRC, RELA, VEGFA, and TP53, while additional nodes were connected indirectly through disease pathways (Figure 4A). Over-represented functions comprised regulation of RNA polymerase II-dependent transcription, apoptosis/anoikis, and phosphorylation, all consistent with processes implicated in hyperthyroidism. Similarly, the subnetwork for *Veratri Rhizoma Et Radix* showed resveratrol and diosgenin directly connected to TGFB1, TNF, and CAT, with downstream influence on phosphorylation, RNA polymerase II-dependent and DNA-templated transcription, gene-expression regulation, and apoptotic programs (Figure 4B).

**Figure 5:**
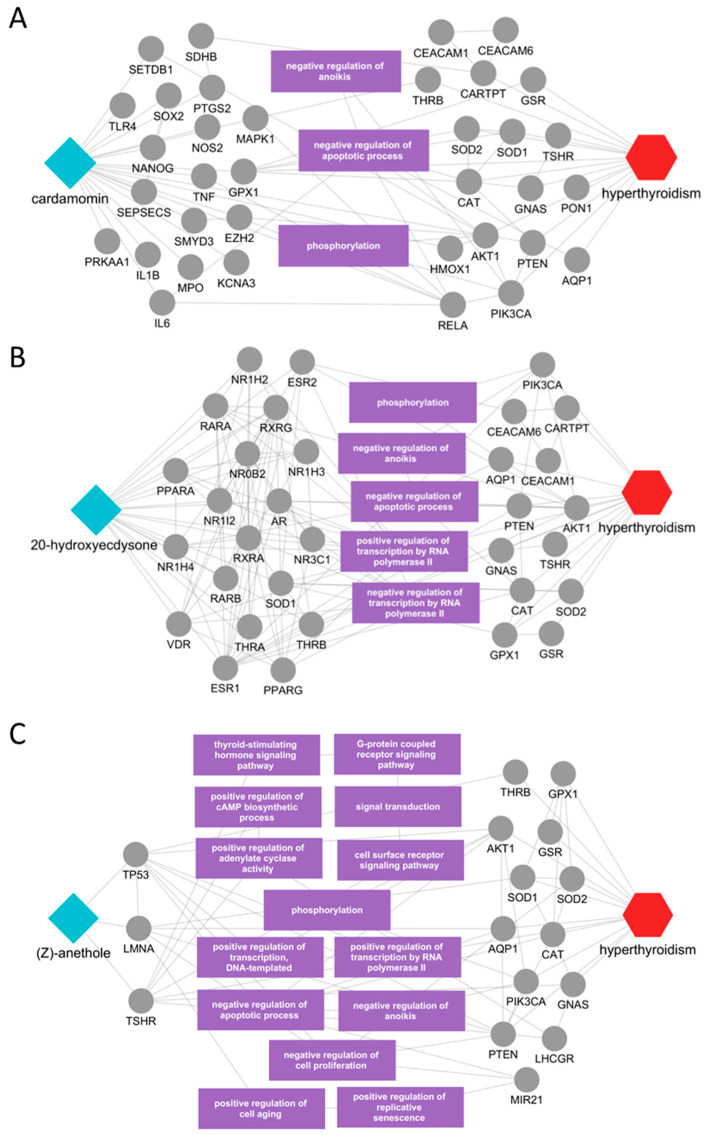
Key mechanisms of compounds without prior disease evidence in hyperthyroidism. (**A**) cardamomin (*Alpiniae Katsumadai Semen*); (**B**) 20-hydroxyecdysone (*Achyranthis Radix*); (**C**) (Z)-anethole (*Illici Veri Fructus*). Each disease-focused subnetwork links the compound to putative disease-relevant protein targets and enriched biological processes. Cyan diamonds represent compounds, gray circles represent protein targets, purple rectangles indicate enriched biological processes/pathways, and the red hexagon denotes the disease (hyperthyroidism).

We then examined compounds without prior disease evidence. The subnetwork for cardamomin (*Alpiniae Katsumadai Semen*) showed direct connections to THRB, TSHR, GNAS, TNF, IL6, IL1B, and CAT, and interactions with additional regulators (MAPK1, AKT1, PTEN, RELA) involved in anoikis, apoptosis, and phosphorylation (Figure 5A). 20-Hydroxyecdysone (*Achyranthis Radix*) interacted directly with THRB, TSHR, GNAS, and CAT, and propagated to disease-relevant functional modules, suggesting roles in phosphorylation, anoikis, and apoptosis (Figure 5B). Also, (Z)-anethole (*Illici Veri Fructus*) linked directly to THRB, TSHR, GNAS, and CAT, with secondary interactions involving PIK3CA, AKT1, PTEN, and TP53; these nodes participate in TSH signaling, GPCR-cAMP pathways (including adenylate cyclase activity), and transcriptional control, as well as apoptosis/anoikis and phosphorylation (Figure 5C). Collectively, these subnetworks highlight plausible molecular interactions that support the therapeutic potential of the nominated compounds and provide testable mechanistic hypotheses.

## 3. Discussion

This study employed a multiscale network with a biased random-walk-based analysis to nominate candidate herbs and active compounds for hyperthyroidism and to probe their putative mechanisms of action. Among the top 10 herbs showing high correlation scores with the disease diffusion profile and statistically significant overlap with disease targets (*p*-value < 0.05) were *Geranii Herba*, Laminaria (Kombu), Gastrodiae Rhizoma, Veratri Rhizoma, *Alpiniae Katsumadai Semen*, and *Achyranthis Radix*. Of these, *Geranii Herba* and Veratri Rhizoma have reported evidence supporting efficacy in hyperthyroidism, whereas Laminaria, Gastrodiae Rhizoma, *Alpiniae Katsumadai Semen*, and *Achyranthis Radix* have limited or no prior evidence and thus emerge here as novel candidates. These findings deepen our understanding of the effects of multi-component traditional remedies on hyperthyroidism and provide a basis for therapeutic discovery.

The multiscale network approach enabled evaluation of broad drug–disease impacts in the human interactome, extending beyond direct protein–protein interactions to incorporate biological functions and pathways [23]. Prior work suggests that this strategy can outperform alternative network-based methods in recovering disease-relevant herbal actives and mechanisms [24]. We simulated diffusion with a biased random walk and computed interaction similarity. This improved predictive performance by shifting transition probabilities toward biological-function nodes rather than solely protein nodes. This allowed us to capture how herb targets influence core pathways and mechanisms at a systems level. Previously applied to predict the therapeutic effects of polyphenols in oxidative liver injury, the framework was here extended to hyperthyroidism, successfully identifying candidate herbs and core protein targets that impinge on disease-salient pathways.

Using enrichment analysis, we delineated signaling pathways and biological functions associated with core targets of the prioritized herbs. KEGG results highlighted MAPK and HIF-1 signaling—axes linked to inflammation and cell-death control—as well as the thyroid-specific thyroid hormone signaling pathway. In Graves’ disease, a prototypic inflammatory autoimmune subtype of hyperthyroidism, systemic inflammatory markers (e.g., hs-CRP, IL-6) have been reported to increase in the hyperthyroid state and decline after treatment [6]. Apoptosis governed by the Fas/FasL system is repeatedly observed in Graves’ disease; both thyrocytes and infiltrating lymphocytes undergo Fas/FasL-mediated apoptosis, and IL-1β can modulate this pathway [25]. Consistent with these observations, PI3K–Akt and p53 pathways were among the top enriched routes, underscoring the importance of apoptosis in hyperthyroidism. In addition, several of the enriched signaling axes—including PI3K–AKT, MAPK, and HIF-1—are central regulators of oxidative stress responses. This suggests that the identified herbal components may help restore cellular homeostasis by normalizing oxidative stress reactions and modulating downstream signaling cascades associated with inflammation, apoptosis, and thyroid hormone activity. The parathyroid hormone synthesis, secretion, and action pathway also emerged, aligning with the central role of calcium–phosphate homeostasis in bone metabolism; patients with Graves’ disease often present with reduced bone mineral density at diagnosis that may normalize after treatment [26,27,28]. As a disease-specific axis, the thyroid hormone signaling pathway directly captures hormone synthesis and action.

GO enrichment further supported associations with apoptotic regulation and inflammatory-response modulation, processes closely tied to disease progression in Graves’ disease. Target proteins localized predominantly to the nucleus (including transcriptional regulator complexes), mitochondria, endoplasmic reticulum, and peroxisomes, consistent with roles in intracellular metabolism, protein synthesis/folding, and redox control. In the Molecular Function category, enrichment for transcription-factor binding and regulation of RNA polymerase II-dependent transcription suggests the potential to reshape gene-expression programs. Thyroid hormones are principal regulators of RNA polymerase II-driven transcription; in states of hormone excess, global expression patterns are reorganized, with metabolic, growth, and cell-death pathways becoming aberrantly activated [29]. Together, these results imply that the therapeutic effects of high-scoring herbs may be mediated via core targets that sit at the intersection of diverse signaling routes and biological functions.

Beyond previously supported herbs (*Geranii Herba*, Veratri Rhizoma), our analysis nominated *Alpiniae Katsumadai Semen*, *Achyranthis Radix*, and *Illici Veri Fructus* as under-recognized candidates. Pharmacological studies report anti-inflammatory, antioxidant, antitumor, and antimicrobial activities for *Alpiniae Katsumadai Semen* [30,31,32]; anti-inflammatory, osteogenic (bone-metabolism-modulating), neuroprotective, and immunomodulatory activities for *Achyranthis Radix* [33,34,35,36]; broad antibacterial, antioxidant, anti-inflammatory, antiviral, analgesic, and anxiolytic/CNS-depressant (sedative-like) effects for *Illici Veri Fructus* [37,38]. In line with these properties, our multiscale analysis showed significant overlap between their active–compound targets and hyperthyroidism diffusion profiles, supporting therapeutic relevance.

Compound-centric subnetworks provided mechanistic resolution. For *Geranii Herba* and Veratri Rhizoma, our network linked ellagic acid to PI3K–AKT/MAPK and growth-factor signaling nodes; ellagic acid has been demonstrated in in vivo mouse tumor models to suppress VEGF/VEGFR2 signaling and downstream PI3K–AKT/MAPK cascades, consistent with its role in phosphorylation and apoptosis regulation [39]. For resveratrol and diosgenin, the predicted connections to TGFB1/TNF/CAT align with in vitro and in vivo findings showing that resveratrol inhibits TNF-α–NF-κB signaling, modulates TGF-β1/Smad pathways, and up-regulates catalase; diosgenin likewise dampens TNF-α–NF-κB signaling [40]. Among newly highlighted actives, cardamonin (*Alpiniae Katsumadai Semen*) has been validated in mouse inflammatory models, where it reduced TNF-α/IL-1β/IL-6 and inhibited NF-κB/MAPK signaling, supporting our network-derived links to MAPK1/AKT1/PTEN/RELA and related apoptosis/phosphorylation nodes [41]. For 20-hydroxyecdysone (*Achyranthis Radix*), direct engagement of thyroid hormone receptors in humans has not been demonstrated; however, 20E exerts ERβ-dependent and PI3K/AKT-intersecting actions (vasodilation, anabolic signaling) that have been observed primarily in ex vivo ovine muscle arterioles and in vitro human endothelial cell studies, consistent with the predicted regulation of transcriptional programs via nuclear-receptor/kinase crosstalk [42]. For (Z)-anethole (*Illici Veri Fructus*), evidence from human feto-placental in vitro co-culture models indicates that trans-anethole enhances cAMP and activates PKA/PKC signaling to promote steroidogenesis [43]. However, no direct evidence currently links (Z)-anethole or *Illici Veri Fructus* to thyroid hormone regulation, suggesting that this network-based prediction represents a novel mechanistic hypothesis. Collectively, these literature-supported links keep our mechanistic hypotheses compact: convergence on PI3K–AKT/MAPK, NF-κB-driven inflammation, cAMP-dependent signaling, and RNA polymerase II-linked transcriptional control.

Despite the strong network-level interpretability of the multiscale interactome, this study is inherently in silico and thus subject to several limitations. First, the model does not account for pharmacokinetic parameters such as the absorption, metabolism, and bioavailability of individual ingredients, which may substantially influence their in vivo efficacy. Second, the composition of herbal extracts varies depending on origin, harvesting, and extraction methods, introducing variability not captured by the curated databases. Third, although the biased random-walk algorithm provides mechanistic hypotheses by connecting herbs, ingredients, and disease targets, experimental and clinical validation remains essential to confirm the predicted associations and safety profiles. In addition, demographic and physiological factors such as age, sex, comorbidities, and hormonal status were not considered in this network-based model. These factors may modulate thyroid hormone metabolism, immune responses, and drug sensitivity, and should therefore be incorporated in future translational and clinical investigations. Future work integrating pharmacokinetic modeling, standardized extract data, and in vitro or in vivo assays will be required to overcome these translational gaps.

## 4. Materials and Methods

An herb–ingredient–target network was constructed by integrating curated herb–ingredient associations with experimentally supported ingredient–target interactions, with low-frequency nodes excluded to ensure robustness (Section 4.1). Target sets were subsequently interpreted by gene set enrichment analysis to identify implicated biological functions and pathways relevant to the disease context (Section 4.2). Disease-related genes were then mapped onto a multiscale interactome, and biased random-walk diffusion profiles were computed to quantify ingredient–disease proximity and infer putative mechanisms and candidate herbs and ingredients (Section 4.3 and Section 4.4).

### 4.1. Herb-Ingredient-Target Network Construction

Herbs and their ingredient data were obtained from the OASIS traditional medicine database (https://oasis.kiom.re.kr/index.jsp, (accessed on 21 August 2024)), which is maintained by the Korean Institute of Oriental Medicine (KIOM). The OASIS platform includes data on potential active ingredients identified through physicochemical analysis techniques such as HPLC and UPLC and validated by experts in pharmacology and traditional medicine. In this study, 12,871 associations involving 420 herbs and 4786 ingredients were collected, with each ingredient identified by its PubChem CID. These data were utilized as the foundational input for the network analysis. Experimentally validated ingredient–target interaction data were compiled from authoritative databases, including DrugBank 5.0 [44], Therapeutic Target Database (TTD 2.0) [45], and the Search Tool for Interactions of Chemicals (STITCH 5) [46], which provide extensive information on established and potential targets, associated diseases, biological pathways, and drugs targeting these proteins.

A network was then constructed to illustrate the relationships between herbs, ingredients, and protein targets. Nodes in the network represented herbs, ingredients, and protein targets, while edges indicated herb–ingredient or ingredient–target interactions. All edges were unweighted and undirected, reflecting the presence of interactions without implying directionality or strength. Ingredients identified through PubChem CID were cross-referenced and integrated with the ingredient–target data. Herbs with fewer than three target-associated ingredients were excluded to maintain robust and reliable network analysis. This threshold was implemented because herbs with at least three active components are more likely to exhibit significant pharmacological effects and provide reliable interaction data. The resulting network allowed the visualization and analysis of interactions among herbs, components, and protein targets. For each herb, the simple pathway count was calculated, taking into account instances where multiple components influenced a single target. This approach enabled the selection of the top 50 targets, with each target’s relative importance assessed accordingly.

### 4.2. Enrichment Analysis

Biological processes and signaling pathways associated with protein targets were analyzed using gene set enrichment analysis (GSEA) with the GSEApy module (version 1.1.3) in a Python 3.7.0 environment, facilitated by the Enrichr platform (http://amp.pharm.mssm.edu/Enrichr/, (accessed on 12 September 2024)) [47,48]. Enrichr draws on diverse gene-set libraries, including Gene Ontology and Kyoto Encyclopedia of Genes and Genomes (KEGG) [49,50], to perform enrichment analysis. *p*-values, z-scores, and combined scores were calculated to evaluate the signaling pathways and biological functions related to herbal ingredient targets. The combined score, derived by multiplying the logarithm of the *p*-value with the z-score, ensured a systematic evaluation of the effects of herbal components on specific biological pathways. Signaling pathways associated with hyperthyroidism were defined as the pathways below as defined in KEGG: MAPK signaling pathway, Thyroid hormone signaling pathway, Calcium signaling pathway, HIF-1 signaling pathway, p53 signaling pathway, mTOR signaling pathway, PI3K-Akt signaling pathway, Wnt signaling pathway, and Parathyroid hormone synthesis, secretion and action.

### 4.3. Disease Related Targets

Hyperthyroidism-related protein data were curated from DisGeNet (https://www.disgenet.org/, (accessed on 12 September 2024)), a database that meticulously maps disease–gene associations to ensure high reliability [51]. This study focused exclusively on expert-reviewed disease–gene associations provided by DisGeNet for “Hyperthyroidism” to maintain relevance to the research. Data in this curated set were drawn from reputable sources, such as UniProt, the Comparative Toxicogenomics Database, Orphanet, Clinical Genome Resource, Genomics England PanelApp, Cancer Genome Interpreter, and the Psychiatric Disorders Gene Association Network. Disease–gene associations based on homology in animal models or derived from computational literature mining, as well as those labeled as therapeutic, were excluded. The refined disease–protein interaction network served as the basis for validating hyperthyroidism-related protein data in this study.

### 4.4. Multiscale Network Analysis for Predicting Disease Associations

A multiscale interactome was constructed based on the methodology of Ruiz et al., which integrated three types of interactions: protein–protein, protein–biological function, and biological function–function interactions [23]. Human protein–protein interaction data were obtained from sources such as the Biological General Repository for Interaction Datasets (BioGRID 3.5.178), the Database of Interacting Proteins (DIP), and the Human Reference Protein Interactome Mapping Project (HuRI), encompassing 387,626 physical interactions among 17,660 proteins. Protein–biological function interactions were extracted from the human Gene Ontology database, encompassing 34,777 experimentally verified associations between 7993 proteins and 6387 biological functions. Biological function–function interactions were organized into a hierarchical network with 22,545 associations among 9798 functions. Diffusion profiles were computed using the multiscale interactome to evaluate the propagation effects of herbal ingredients on proteins associated with hyperthyroidism. This analysis applied a biased random walk with a restart algorithm to quantitatively assess the influence of herbal targets and ingredient targets on hyperthyroidism-related proteins. Correlation scores were then calculated between herb–ingredient profiles and disease profiles, enabling the identification of promising herbal candidates and ingredients for treating hyperthyroidism. For each ingredient–disease pair, primary mechanisms were identified by analyzing diffusion profiles and selecting the top k-proteins or biological functions influenced by the ingredient or the disease. A network was constructed to highlight the significant relationships. Ingredients not associated with disease-related proteins or biological functions were excluded. The top-ranking entity in the diffusion profile was considered the most critical for treatment based on its substantial influence. The parameter k was set to 20 in this analysis to ensure thorough exploration of influential nodes. Previous research indicated that setting k to 10 captured approximately 50% of the total visitation frequency in the diffusion profile. Increasing k to 20 allowed a greater proportion of visitation frequency to be captured, improving the analysis’s comprehensiveness. For further details on diffusion profile calculations, including mathematical formulas, iterative processes, and parameter selection rationale, refer to prior research [23].

## 5. Conclusions

In conclusion, we prioritized herbs and ingredients relevant to hyperthyroidism and mapped their putative mechanisms. The framework recovered known thyroid-modulating herbs and highlighted underrecognized candidates whose targets converge on MAPK, PI3K–AKT, p53, HIF-1, and thyroid hormone signaling, alongside apoptosis/anoikis and RNA polymerase II-dependent transcription. Compound-centric subnetworks yielded testable hypotheses—particularly for resveratrol, cardamomin, 20-hydroxyecdysone, and (Z)-anethole—linking them to phosphorylation and GPCR–cAMP/TSH pathways. Experimental validation and safety/efficacy studies are now needed to translate these leads. More broadly, this strategy provides a reproducible route to discover multi-target therapeutics for endocrine disease.

## Figures and Tables

**Table 1:** Top 10 ranked herbs with correlation with hyperthyroidism diffusion profile.

Herb Name (Latin)	Correlation Score ^†^	Overlap (*p*-Value ^#^)	Enrichment	References (PMID)
*Geranii Herba* *	0.021	2/50 (6.0 × 10^−3^)	16.91	-
*Veratri Rhizoma Et Radix* *	0.011	5/50 (6.6 × 10^−8^)	42.27	
*Laminariae Japonicae Thallus*	0.010	2/27 (1.7 × 10^−3^)	31.31	18689954 [17] 21975053 [18]
*Alpiniae Katsumadai Semen* *	0.010	2/26 (1.6 × 10^−3^)	32.52	-
*Achyranthis Radix* *	0.010	2/38 (3.5 × 10^−3^)	22.25	-
*Illici Veri Fructus* *	0.010	1/11 (2.6 × 10^−2^)	38.43	-
*Ligustici Tenuissimi Rhizoma et Radix*	0.010	4/50 (4.2 × 10^−6^)	33.82	-
*Mori Folium*	0.010	4/50 (4.2 × 10^−6^)	33.82	-
*Pini Koraiensis Semen*	0.010	4/50 (4.2 × 10^−6^)	33.82	-
*Arecae Semen*	0.009	3/50 (0.2 × 10^−3^)	25.36	29278926 [19]

^†^ The correlation score denotes the correlation score between the disease and herb diffusion profiles. An * next to the herb name marks candidate herbs strongly associated with hyperthyroidism that have not yet been investigated. The ^#^ symbol next to the *p*-value indicates values obtained using the hypergeometric test, applied to evaluate the significance of overlap between datasets.

**Table 2:** KEGG Signaling Pathway Enrichment Analysis of Core Protein Targets.

Term	Overlap	Adjusted *p*-Value	Combined Score	Genes
MAPK signaling pathway	8/294	3.3 × 10^−8^	351.17	*JUN*; *IL1B*; *CASP3*; *AKT1*; *MAPK1*; *TP53*; *TNF*; *RELA*
Thyroid hormone signaling pathway	6/121	6.1 × 10^−8^	593.35	*CCND1*; *BAD*; *AKT1*; *MAPK1*; *ESR1*; *TP53*
Calcium signaling pathway	3/240	8.4 × 10^−3^	37.28	*ADORA2A*; *NOS2*; *NOS3*
HIF-1 signaling pathway	8/109	1.2 × 10^−11^	1463.04	*IL6*; *CDKN1A*; *NOS2*; *NOS3*; *BCL2*; *AKT1*; *MAPK1*; *RELA*
p53 signaling pathway	7/73	4.0 × 10^−11^	1804.99	*CDKN1A*; *CCNB1*; *CCND1*; *CASP3*; *BCL2*; *BAX*; *TP53*
mTOR signaling pathway	4/154	1.5 × 10^−4^	150.43	*PRKAA1*; *AKT1*; *MAPK1*; *TNF*
PI3K-Akt signaling pathway	11/354	1.4 × 10^−11^	657.11	*IL6*; *CDKN1A*; *PRKAA1*; *CCND1*; *NOS3*; *BAD*; *BCL2*; *AKT1*; *MAPK1*; *TP53*; *RELA*
Wnt signaling pathway	4/166	2.0 × 10^−4^	134.66	*JUN*; *CCND1*; *TP53*; *PPARD*
Parathyroid hormone synthesis, secretion and action	3/106	8.4 × 10^−4^	128.09	*CDKN1A*; *BCL2*; *MAPK1*

**Table 3:** Representative ingredients of candidate herbs and their association with hyperthyroidism.

Name	PubChem Compound ID	Correlation Score	Overlap (*p*-Value ^#^)	References (PMID)
***Geranii Herba***				
ellagic acid	5281855	0.007	2/107 (1.4 × 10^−2^)	6724503 [20]
***Veratri Rhizoma Et Radix***				
resveratrol *	445154	0.013	8/326 (1.2 × 10^−8^)	-
diosgenin	99474	0.011	3/25 (8.6 × 10^−6^)	35140607 [21] 28407664 [22]
***Alpiniae Katsumadai Semen***				
cardamomin *	641785	0.012	2/22 (6.0 × 10^−4^)	-
***Achyranthis Radix***				
20-hydroxyecdysone *	5459840	0.009	2/37 (1.7 × 10^−3^)	-
***Illici Veri Fructus***				
(Z)-anethole *	1549040	0.025	1/3 (5.0 × 10^−3^)	-

The ^#^ denotes *p*-values calculated via the hypergeometric test to assess dataset overlap significance. * indicates active ingredients with strong associations to hyperthyroidism that remain uninvestigated.

## Data Availability

The data supporting the findings of this study are included within the manuscript.
